# Psychological and social interventions for mental health issues and disorders in Southeast Asia: a systematic review

**DOI:** 10.1186/s13033-021-00482-y

**Published:** 2021-06-05

**Authors:** Alan Maddock, Carolyn Blair, Nil Ean, Paul Best

**Affiliations:** 1 School of Social Sciences, Education and Social Work, Queen’s University Belfast, Northern Ireland, Belfast, UK; 2 Department of Psychology, Royal University of Phnom Penh, Phnom Penh, Cambodia

**Keywords:** Mental health, Disorder, Psychological and social, Intervention, Mental health system, Review

## Abstract

**Background:**

Mental health issues and disorders are major public health challenges, particularly in low- and middle-income countries in Southeast Asia, where chronic shortages in mental health services and human resources exist. The development of effective and accessible mental health systems in Southeast Asia will require evidence based psychological and social interventions. This systematic review provides a narrative synthesis of the evidence on the effectiveness of such interventions for mental health issues and disorders in Southeast Asia.

**Methods:**

A comprehensive literature search of 7 electronic databases (PsycINFO, Medline (Ovid), Cochrane library, EMBASE, SCOPUS, APA PsycArticles, and Social Care Online) was undertaken.

**Results:**

Thirty two studies employing RCT designs to evaluate the effectiveness of a range of psychological and social mental health interventions on a number of different mental health outcomes were included in this review. The disparate intervention programmes reviewed were categorised as: lay delivered, yoga, aerobic and/or meditation based, cognitive behavioural therapy oriented, eye movement desensitization and reprocessing based (EMDR), health worker delivered, and hybrid programmes. The majority of the studies included in this review were of low to moderate quality due to the variability in the quality of the study design. The highest quality, and most promising evidence came from the evaluations of lay delivered interventions. This evidence demonstrates the feasibility and potential sustainability of implementing such interventions in resource constrained contexts.

**Conclusions:**

The review findings indicate that a disparate array of mental health interventions can be implemented effectively in a range of Southeast Asian mental health and health settings. There is a clear need for significantly more research however, through higher quality and larger scale RCTs before it will be known more definitively, if these interventions are effective, and for whom they are most effective in different Southeast Asian contexts.

**Supplementary Information:**

The online version contains supplementary material available at 10.1186/s13033-021-00482-y.

## Background

It has been estimated that there are approximately 322 million people with a depressive disorder (4.4% population prevalence) and 254 million people with an anxiety disorder (3.6% population prevalence) worldwide [1]. Five types of mental illness were reported in the top twenty causes of the global burden of disease in 2013 [2]. These were major depression (ranked 2nd), anxiety disorders (ranked 7th), schizophrenia (ranked 11th), dysthymia (ranked 16th) and bipolar disorder (ranked 17th) [2]. Mental health disorders are not only a major public health challenge but they are also an impediment to economic development in low- and middle-income countries (LMIC) [3]. The WHO Southeast Asia Region comprises of 12 countries (Bangladesh, Bhutan, Democratic People’s Republic of Korea, India, Indonesia, Maldives, Myanmar, Nepal, Sri-Lanka, Thailand and Timor-Leste as member states with Cambodia containing WHO regional country office) and contributes one quarter of the world’s population [3]. Most of the countries in the region belong to the low-income group based on World Bank criteria, and face significant mental health challenges [4, 5] e.g. the estimated depression prevalence rate in Cambodia is five times higher than the worldwide average [6]. The estimated population prevalence rates of depressive and anxiety disorder cases in the WHO Southeast Asia region is 27% and 23% respectively [1]. The rates of mental health disorders in Southeast Asia are typically under-reported due to data scarcity, and a significantly more limited focus on the treatment of mental health disorders in these countries [7]. Mental health disorders typically place a significant psychological, social and economic burden on Southeast Asian citizens, however most patients receive no treatment [7]. This treatment gap is particularly apparent in the lowest-resource settings, where it can be as high as 90% [7].

The majority of Southeast Asian countries have national mental health policies, however their effective implementation has proved to be hugely challenging [5]. This appears to be due to the limited allocation of government health funding to mental health in the region, averaging at approximately 2%, ranging from between 0.06% in India to 4% in Thailand [4, 8–11]. The vast majority of this allocation, approximately 80–90%, goes directly to mental health hospital service provisions, which are generally concentrated in urban areas, and are inaccessible or unaffordable to rural populations [4, 5, 8, 9, 11–13]. The limited government funding allocations have led to chronic shortages in mental health services and trained mental health professionals in the region [5, 12]. The median number of professionals working in the field of mental health in the region is 5.3 per 100,000 population, which is about half of the overall global median [11]. A significant majority of countries in the region have less than one psychiatrist per 100,000 population. In terms of availability of psychiatric beds across all facilities, the Southeast Asia Region (0.23/100,000 population) falls well below the global median (3.2/100,000 population) [5, 7, 13].

Good population mental health is central to achieving UN Sustainable Development Goals (e.g. Goal 3, Good Health and Well-being; Goal 8, Decent Work and Economic Growth) [14]. The gaps between research, public health priorities and clinical mental health practice have consistently been identified as a significant challenge to achieving good population mental health by researchers, policymakers and clinicians worldwide [10]. These historical gaps are especially wide in LMICs, such as those in Southeast Asia, where evidence-based information is scarce, and research capacity building is limited [15, 16]. It has also been consistently suggested that policymakers in Southeast Asian countries should facilitate training in the field of mental health, improving access to evidence based psychological and social interventions, with the aim of integrating mental health services within primary health care [3, 4, 17]. This process would likely close mental health treatment gaps, enhance access to services, increase cost effectiveness and generate improved health and mental health outcomes [3–5]. In the absence of appropriate numbers of mental health professionals and mental health funding allocations, in order to achieve these goals, particular attention will need to be paid to generating innovative solutions for long-term complex health and mental health issues [4], solutions that are not necessarily imported from elsewhere but are developed within the contexts in which they will operate [4]. It remains unclear which mental health psychological and social interventions are the most effective in Southeast Asian contexts [18], with a number of international services being criticized for being culturally insensitive and lacking integration with local communities [19]. Most psychological and social mental health interventions e.g. Cognitive Behavioural Therapy (CBT), have been developed in high-income countries with western cultures and may have reduced effectiveness in Southeast Asia because emotional distress is experienced and communicated differently in different social contexts [20, 21]. The development of effective and accessible mental health systems in Southeast Asia will thus require evidence-based culturally appropriate psychological and social interventions [4]. Thus, an urgent task in addressing the mental health of Southeast Asian countries should be on improving and expanding the evidence base on what potentially effective interventions might be, particularly in resource-constrained countries [3]. Providing evidence of this nature is likely to support the development of enhanced evidence-based health policies and practice in Southeast Asia and improve the capacity of local governments and large scale non-governmental organisations (NGOs) to address the needs of people with mental health issues and disorders [3, 22–24]. Identifying these interventions, particularly those that may be cost effective might also help to convince authorities in Southeast Asia to implement the policy that is available more thoroughly [3].

This systematic review aims to (1) identify and systematically summarise the best available evidence on the effectiveness of psychological and social interventions or programmes for mental health issues or disorders in South East Asia, (2) identify gaps in the existing evidence and highlight areas where further research is needed, and (3) identify how future trials should be designed to provide more rigorous evidence.

## Methods

This systematic review was conducted following the general principles published by the NHS Centre for Reviews and Dissemination [25] and reported according to the PRISMA (Preferred Reporting Items for Systematic Reviews and Meta-Analyses) guidelines [26].

### Search strategy

The electronic search of seven databases (PsycINFO, Medline (Ovid), Cochrane library, EMBASE, SCOPUS, APA PsycArticles and Social Care Online) was undertaken in May 2020. The search strategy varied across the databases, but the same keywords applied throughout.

### Keywords

("Post trauma*" or "Post-trauma*" or PTSD or "Psych* disorder" or "mental health disorder" or “mental health issue” or Depress* or Anxiet* or "Psych* illness" or "Psych* disorder" or Disabilit* or "Mood disorder" or "Personality disorder" or "Neurological impairment" or "cognitive impairment" or "Substance use disorder*") ab.

### AND

("social support" or "mind–body therapy" or "Dialectical behavioural therapy" or "Acceptance and commitment therapy" or "Peer support" or "Mental Health Services" or CBT or "Cognitive Behavioural therapy" or "Cognitive Behavioral therapy"OR Biofeedback or "Breathing Exercise" or hypnosis or imagery or meditation or mindfulness or Psychodrama or "Tai Ji" or neurofeedback or Yoga or "therapeutic touch" or Aromatherapy or Bibliotherapy or Counsel* or "Crisis Intervention" or trauma-focus* or "narrative therapy" or "person-centred therapy" or "integrative therapy" or "Humanistic therapy" or "Eye Movement Desensitisation and Reprocessing" or EMDR or psychotherap* or psycho-therap*).ab.

### AND

("Southeast Asia" or "South East Asia" or "South-east Asia" or Indonesia or "Sri Lanka" or Thailand or Timor-Leste or Cambodia or Bangladesh or Bhutan or "Democratic People’s Republic of Korea" or India or Maldives or Myanmar or Nepal).ab.

### AND

(Intervent* or program* or therap* or treat*).ab.

### Study selection and data extraction

The results of all search strategies for the interventions component of the review were imported to a screening tool [27]. After removing duplicates, the titles and abstracts were screened independently by AM and CB, with the aim of identifying potentially relevant studies. During this phase, inclusion and exclusion criteria (see Table 1) were applied and disagreement was resolved through discussion. Subsequently, full texts of the promising studies were obtained and their reference lists were examined by AM, CB, NE and PB. For the purpose of this review, a mental health psychological or social intervention was defined as any planned action, programme or policy, which was undertaken with the aim of improving mental health issues or disorders though the improvement of psychological or social factors rather than biological ones [28]. This definition allowed the inclusion of purely psychological interventions, as well as interventions which focus on developing the person’s social environment with a view to enhancing their mental health e.g. through the development of social supports. Interventions which have been deemed generally to be physiological in nature but included a psychological and/or social focus (e.g. yoga) were also included [28]. A counselling intervention was deemed to involve a purposeful conversation(s) arising between one person (or group) with the intention of reflecting on and resolving a problem of living, and another person with a willingness to assist in that endeavour [29]. A therapy intervention represents a deeper, more fundamental level of focussed work, over a longer period, usually with clients with a higher prevalence of mental health issues or disorders [29]. Psychoeducation was deemed to be any form of training targeted at promoting awareness and providing tools to manage, cope and live with a mental health issue or disorder [30]. Behavioural activation was deemed to be a low intensity intervention, based on the principles of operant conditioning through scheduling to encourage depressed people to re-engage with potential sources of positive reinforcement, in setting task-focused goals, in order to diminish avoidance and patterns of negative reinforcement [31, 32]. Cognitive behavioural therapy (CBT) was deemed to be a defined form of psychotherapeutic treatment which helped people to learn how to identify and change disturbing or destructive thought patterns that had a negative impact on emotions and behaviour [33]. Eye movement desensitisation and reprocessing (EMDR) was deemed to be a defined therapy which required patients to safely retrieve a traumatic memory, with the support of a trained therapist, in order to reduce the emotional distress that this recollection created, through the reappraising and reprocessing of the thoughts associated with the events of the traumatic experience [34].

**Table 1:** Inclusion/exclusion criteria for the review

	Inclusion criteria	Exclusion criteria
Types of trials	Any randomised control trial (RCT) reported in English examining the effectiveness of psychological and social interventions or treatments of mental health issues or disorders in Southeast Asia	Case–control trials, cohort trials, cross-sectional trials, case reports, series and qualitative trials
Types of publications	Published trials conducted in Southeast Asia and reported in English	Not based in Southeast Asia Non-published trials and dissertations
Types of participants	Adults, 18 years and older with mental health issues or disorders	Children and healthy people
Types of interventions	Any psychological and social interventions	
Types of outcomes	Any mental health issue or disorder	
Types of comparators	Any comparator. This might include inactive control such as treatment as usual (TAU) and waiting list or active group, such as antidepressants or other psychological interventions	

A data extraction tool was developed in order to for the characteristics of studies using the PICOS (Population, Intervention, Comparator, Outcomes and Study design) framework to be collected [35]. Data extraction was conducted by CB and checked by AM.

### Quality appraisal

The quality of the methodologies undertaken in each study was assessed using the Cochrane risk of bias tool [36, 37]. This quality assessment tool contains seven domains which assess the risk of biased findings due to: sequence generation, allocation concealment, blinding of participants and personnel, outcome assessment, incomplete outcome data, selective reporting and other biases. The risk of bias quality appraisals were conducted by AM, CB, NE and PB.

### Data analysis and synthesis

The original intention for the data analysis and synthesis in this review was for a meta-analysis per outcome to be conducted should there be satisfactory clinical and statistical homogeneity. When all the included RCTs had been reviewed, it was clear that there was too much heterogeneity (of populations and interventions examined, research methods employed, and outcomes measured) for a sound meta-analysis to be conducted. Therefore, to ensure that the results could be reported in a systematic manner, a modified narrative synthesis was conducted [38] using an adapted version of the International Classification of Health Interventions (ICHI) [39]. The ICHI is a common tool used for reporting and analysing health interventions for statistical and quality purposes. This is an international standard that enables comparison of data between countries and services [39]. The ICHI uses three axes to describe interventions: (1) Target: the entity on which the Action is carried out; (2) Action: the deed done by an actor to the Target, and (3) Means: the processes and methods by which the Action is carried out [39]. This modified narrative synthesis involved: (1) developing a preliminary synthesis, through organising and categorising the studies in terms of (a) target group and the mental health outcomes focussed on, (b) the action or type of intervention, and c) the means or methods of the intervention e.g. was it group or individual based? and how long was the intervention for?; (2) exploring relationships in the data, i.e. considering factors that may explain any similarities or differences in the nature of the effects in the included studies, and (3) assessing the robustness of the synthesis, i.e. through assessing the strength of the evidence for each intervention type [38].

### Results

**Fig. 1:**
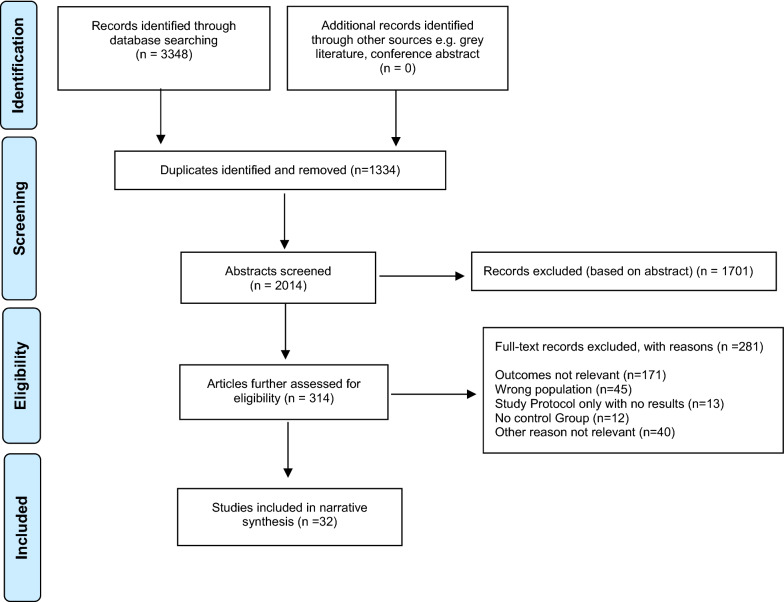
*PRISMA Flow Diagram* [26]

The electronic searches of seven databases retrieved 3348 titles and abstracts. After adjusting for duplicates and reviewing the titles and abstracts, 1334 studies were removed. In the first phase of the screening for eligibility, 2014 abstracts and titles were screened against the inclusion and exclusion criteria, resulting in 1701 studies being excluded as they did not meet the inclusion criteria. In the second phase, 314 were assessed for eligibility, of which 281 records were excluded for the following reasons: outcomes not relevant (n = 171), wrong population (n = 45), study protocol only with no results (n = 13), no control group (n = 12), other reason not relevant (n = 40). Finally, 32 studies met the inclusion criteria of this review (see Fig. 1).

A narrative summary of the reviewed studies including: the intervention modality, outcomes targeted and the nature and duration of the intervention’s delivery along with the results of each intervention’s effectiveness is presented in Table 2 below. Sixteen studies were conducted in India [40–56]. Eight studies were conducted in Thailand [48, 57–63], two in Nepal [64, 65] and two in Cambodia [66, 67]. One was conducted in Indonesia [68], Timor-Leste [69] and Sri Lanka [70]. One study was conducted in Pakistan and India and their results were pooled [71]. Furthermore, two studies presented different parts of their results in different publications: [44, 61–63]. The majority of the studies [18 out of 32] were conducted since 2015.

**Table 2:** Narrative summary of reviewed studies

Paper title (with unique ID)	Outcome(s) targeted	Intervention type	Number of participants	Individual or Group delivery	Duration	Effectiveness (results)
The Healthy Activity Program (HAP), a lay counsellor-delivered brief psychological treatment for severe depression, in primary care in India: a randomised controlled trial. (Patel et al. 2017) [49]	Depression Severity Remission From Depression Suicidal Thoughts	HAP is a brief psychological treatment adapted from behavioural activation including: psychoeducation, behavioural assessment, activity monitoring, activity structuring and scheduling, activation of social networks, and problem solving	N = 493	Individual – at home or over the telephone	6–8 week period for 30–40 min per session	Intervention group (IG) experienced reduced depressive symptom severity (P < 0.001), higher remission in depression (P < 0.001) and reduced suicidal thoughts or attempts (P < 0.0001) versus the control group (CG) after 3 months
Effectiveness of the Thinking Healthy Programme (THP) for perinatal depression delivered through peers: Pooled analysis of two randomized controlled trials in India and Pakistan. (Vanobberghen et al. 2020) [71]	Depression severity Remission from depression	THP is based on: behavioural activation, active listening, collaboration with the patient’s family, guided discovery and homework	N = 850	Both	6–14 weeks over the 6 month prenatal period to six months post-childbirth	Compared to CG the IG was effective at reducing levels of depression (P = 0.03) and remission from depression (P = 0.04) at 6 months
Delivering the Thinking Healthy Programme for perinatal depression through peers: an individually randomised controlled trial in India. (Fuhr et al. 2019) [41]	Depression severity Remission from depression	THP	N = 280	Both	6–14 weeks over the 6 month prenatal period to six months post-childbirth	IG reported reduced depression symptom severity at 3 months (P = 0.01), disability (P = 0.009) relative to the CG. The IG also reported improved recovery (P = 0.03). No evidence of an intervention effect on remission at 3 months (P = 0.08) or WHO-DAS score at 6 months (P = 0.16)
Internet-based behavioural activation with lay counsellor support versus online minimal psychoeducation without support for treatment of depression: a randomised controlled trial in Indonesia (Arjadi et al. 2018) [68]	Depression Fear and avoidance Social Support Quality of Life	Online behavioural activation	N = 313	Individual	Eight weekly structured modules, with assignments, that could be completed in 30–45 min each	IG delivered by lay counsellors with peer support was led to significantly lower depression scores versus CG (P = 0.017). These results were maintained at 3 and 6 months
Effectiveness of psychological treatments for depression and alcohol use disorder delivered by community-based counsellors: two pragmatic randomised controlled trials within primary healthcare in Nepal (Jordans et al. 2019) [64]	Depression Substance Use Disorder	HAP	N = 120	Individual – at home or over the telephone	6–8 week period for 30–40 min per session	IG vrs CG reported significantly lower depressive symptom severity (P < 0.001) at 3 and 12 month follow up (P < 0.001) amongst depressed patients in primary care
Effectiveness of an intervention led by lay health counsellors for depressive and anxiety disorders in primary care in Goa, India (MANAS): a cluster randomised controlled trial. (Patel et al. 2010) [56]	Common Mental Health Disorders Disability Suicidal Thoughts	MANAS is a collaborative stepped-care intervention which includes case management along with psychoeducation and individual counselling (a minimum of 6 sessions with maximum of 12 sessions) provided by a trained lay health counsellor, which were supplemented by antidepressant medication dispensed by a primary care physician and supervised by a mental health specialist	N = 2796	Individual level within clusters	Over a 6 month period	This study conducted a subgroup analysis on participants with depression and no significant difference was found between the IG and CG (P = 0.07)
Lay health worker led intervention for depressive and anxiety disorders in India: impact on clinical and disability outcomes over 12 months. (Patel et al. 2011) [40]	Common Mental Health Disorders Disability Suicidal Thoughts	MANAS	N = 2796	Individual level within clusters	Over a 6 month period	The overall effect was a 30% reduction in prevalence in the ICD-10 diagnosis group between arms (P = 0.02); 24% reduction in the depression subgroup (P = 0.04); 34% reduction in the screen-positive group (P = 0.03); and 57% reduction in the sub-threshold subgroup (P = 0.11). Results not sustained at 6/12 months
5-HTTLPR and MTHFR 677C > T polymorphisms and response to yoga-based lifestyle intervention in major depressive disorder: A randomized active-controlled trial. (Tolahunase, 2018a) [51]	Major Depressive Disorder	Yoga and meditation lifestyle intervention (YMLI): incorporating physical yoga poses, breathing exercises and meditation	N = 178	Mix of both	Each session lasting for 2 h, practiced 5 days a week over 12 weeks. The first 2 weeks of the intervention included supervised group sessions, which included lectures on yoga, meditation, major depressive disorder (MDD) and the importance of depressive symptom prevention and management. The remaining 10 weeks was completed individually at home, including one to one and unsupervised sessions	The IG had a significant effect on depression severity versus CG (P < 0.001). A stratified analysis found that this change was significant for the deemed to have moderate (P = 0.029) and severe MDD (P < 0.01) at baseline but not mild MDD (P < 0.072) after YMLI compared to CG. Clinical improvement was more significant for the women in IG (P = 0.032)
Yoga- And Meditation-Based Lifestyle Intervention Increases Neuroplasticity and Reduces Severity of Major Depressive Disorder: A Randomized Controlled Trial. (Tolahunase et al. 2018b) [42]	Major Depressive Disorder	YMLI	N = 58	Mix of both	Each session lasting for 2 hours, practiced 5 days a week over 12 weeks. The first 2 weeks of the intervention included supervised group sessions, which included lectures on yoga, meditation, major depressive disorder and the importance of depressive symptom prevention and management. The remaining 10 weeks was completed individually at home, including one to one and unsupervised sessions	For the IG there was a significant decrease difference between means in BDI-II (depression) score (P < 0.001) and significant increase in BDNF (Brain-derived neurotrophic factor) (P < 0.001) post 12- weeks compared to the CG
Comparative Effectiveness of Mindfulness-Based Therapy in Sleep Quality of Chronic Insomnia Compared to Standard Cognitive Behavioral Therapy [CBT-I]: A Randomized Controlled Trial. (Siritienthong et al. 2018) [58]	Sleep Quality Depression Anxiety Mindfulness level	Mindfulness Based Therapy	N = 25	Group	Weekly session for 8 weeks lasting 1.5–2 h	There were no differences between the IG and the CG on sleep quality (P = 0.76), depression symptoms (P = 0.49), anxiety symptoms (P = 0.14) and mindfulness levels (P = 0.25)
Effectiveness of integrated body-mind-spirit group intervention on the well-being of Indian patients with depression: a pilot study (Rentala et al. 2013) [43]	Depression Well-being Work and social adjustment	Integrated body-mind-spirit group intervention (IBMSGI): intervention includes lectures on emotional and health management, stress reduction training (including breathing and meditative techniques), acupressure exercises, and homework activities such as writing and drawing, which encourage participant to focus on positive meanings within negative experience	N = 30	Group	Weekly session for 4 weeks lasting for 3 h	Compared with the CG group, the IG group showed statistically significant decreases in depression (P < 0.001) and functional impairment (P < 0.001) and statistically significant increases in the well-being (P < 0.001) over the 6-month interval
Effectiveness of body-mind-spirit intervention on well-being, functional impairment and quality of life among depressive patients—a randomized controlled trial (Rentala et al. 2015) [50]	Depression Well-being Functional impairment Quality of life	IBMSGI	N = 120	Group	Weekly session for 4 weeks lasting for 3 h	Compared with the CG group, the IG group showed statistically significant decreases in depression and functional impairment P < 0.001 and statistically significant increases in the well-being and quality of life P < 0.001 over the 6-month interval
Impact of Yoga Nidra on psychological general well-being in patients with menstrual irregularities: A randomized controlled trials (Rani et al. 2011) [44]	Well-being Anxiety Depression	Yoga Nidra deep relaxation therapy	N = 150	Group	35 min a day, 5 days a week for six months	After six months those in the IG compared with the CG had significant reduction in Anxiety (t-test, P value) 3.00 0.003 Depression 2.57 0.01 and Positive well-being 2.26 0.02
Yoga Nidra as a complementary treatment of anxiety and depressive symptoms in patients with menstrual disorder. (Rani et al. 2012) [52]	Severity of anxiety and depressive symptoms	Yoga Nidra	N = 150	Group	35 min a day, 5 days a week for six months	Compared to the CG the IG improved mild to moderate depression symptoms of women with menstrual disorder (P < 0.02) after 6 months but not severe depressive symptoms. Overall significant improvement in anxiety (P < 0.003) and depression (P < 0.02) in IG in comparison to CG
Psycho-Biological Changes with Add on Yoga Nidra in Patients with Menstrual Disorders: a Randomized Clinical Trial. (Rani et al. 2016) [45]	Anxiety Depression Positive well-being Self-control General health Vitality	Yoga Nidra	N = 87	Group	35 min a day, 5 days a week for six months	IG improved depressive symptoms of women with menstrual disorder (P < 0.02) after 6 months compared to CG. Significant improvement in domains of anxiety (P < 0.01), depression (P < 0.02), positive well-being (P < 0.01), general health (P < 0.04) and vitality (P < 0.02) in IG was noted after six months of yogic intervention when compared to CG
The impact of an add-on video assisted structured aerobic exercise module on mood and somatic symptoms among women with depressive disorders: study from a tertiary care centre in India (Roy, 2018) [46]	Reduction in depression	Video Assisted Structured Aerobic Exercise Program	N = 40	Group	Consists of 20 min of moderate to low intensity aerobic exercises, delivered in a group format via a laptop, supervised by a registered nurse, every day for 10 days	IG versus CG improved levels of depression (P < 0.05) at the programme’s end but not mood score or somatic symptoms related to depression
Cognitive-behavioural therapy for depression among menopausal women: A randomized controlled trial. (Reddy et al. 2019) [47]	Depression	Cognitive Behavioural Therapy (CBT)	N = 102	Group	Weekly sessions for 6 weeks	Compared with the CG, the IG group showed statistically significant decrease in depression scores (P = 0.000) over the 6-month period
A randomized controlled effectiveness trial of cognitive behaviour therapy for post-traumatic stress disorder in terrorist-affected people in Thailand (Bryant et al. 2011) [59]	Reduction in PTSD symptoms	CBT	N = 28	Individual	Weekly sessions for 8 weeks	Compared with CG the IG reported significantly improved PTSD (P = 0.001), depressive symptoms (P = 0.004) and complex grief post treatment symptoms (P = 0.001) post intervention and 3 months (P = 0.007 for PTSD; P = 0.003 for depression and P = 0.003 for complicated grief) post treatment
Evaluation of a bibliotherapy manual for reducing psychological distress in people with depression: a randomized controlled trial. (Songprakun & McCann, 2012a) [61]	Depression Psychological Distress	Bibliotherapy: self-help and CBT based manual	N = 56	Individual	Weekly for 8 weeks	IG versus CG improved depression post treatment (P = 0.018) and 4 weeks (depression P = 0.005) but no change in psychological distress
Effectiveness of a self-help manual on the promotion of resilience in individuals with depression in Thailand: a randomised controlled trial. (Songprakun & McCann, 2012b) [62]	Resilience Depression	Bibliotherapy	N = 56	Individual	Weekly for 8 weeks	IG versus CG improved resilience levels (P = 0.029), and depression post treatment (P = 0.018) and 4 weeks hence (resilience P = 0.004; depression P = 0.005)
Evaluation of a cognitive behavioural self-help manual for reducing depression: a randomized controlled trial (Songprakun & McCann, 2012c) [63]	Depression	Bibliotherapy	N = 56	Individual	Weekly for 8 weeks	IG group showed a significant decrease in depression scores from baseline to post-test (P = 0.018) and from baseline to follow-up (P = 0.005), but no significant decrease from post-test to follow-up (P = 1.00)
Effectiveness of community-based depression intervention programme (ComDIP) to manage women with depression in primary care- randomised control trial. (Indu et al. 2018) [53]	Depression Quality of Life	Community-based depression intervention programme (ComDIP): includes psychoeducation, behavioural activation, relation between negative thoughts and behaviour discussion and drug treatment	N = 60	Individual	Weekly for 8 weeks	In the IG there was a large effect on depressive severity with women with depression (P = 0.01) and quality of life (P = 0.006) was found at 8 weeks versus CG
Effectiveness of non-medical health worker-led counselling on psychological distress: a randomized controlled trial in rural Nepal. (Markkula et al. 2019) [65]	Depression Anxiety	Counselling intervention: emotional support along with problem solving and coping skills development	N = 287	Individual	Five 45 min appointments, two of which were completed in the first week, with meetings being weekly from weeks two to four	IG who received 6 months training, delivered in a primary care setting was effective versus CG at improving depressive and anxiety symptoms at 1 and 6 months (P-Values not reported)
Brief cognitive behavioral therapy for depression among patients with alcohol dependence in Thailand (Thapinta et al. 2014) [48]	Depression Alcohol dependence	CBT	N = 80	Group	6 sessions delivered over 3 weeks	IG delivered over a 3 week period was effective at reducing depression among Thai general hospital patients with alcohol dependence post intervention (P < .01) and 7 weeks later (P < .01)
Effectiveness of a community-based intervention for people with schizophrenia and their caregivers in India (COPSI): a randomised controlled trial (Chatterjee et al. 2019) [55]	Change in Schizophrenia symptoms and disabilities Adherence to antipsychotic treatment and experience of stigma and discrimination	Community care for People with Schizophrenia: individualised treatment plan including clinical review, psychoeducation, medication adherence, health promotion strategies, self-help group referral and community agency engagement	N = 282	Individual	Delivered in different phases: intensive engagement (0-3 months), stabilization (4–7 months) and maintenance phases (8–12 months)	For the IG in the intensive engagement (0-3 months), the stabilization (4–7 months), and the maintenance (8–12 months) vs CG reduced positive and negative symptoms of Schizophrenia and disabilities at 12 months (P = 0.01)
The effect of telephone support on depressive symptoms among HIV-infected pregnant women in Thailand: an embedded mixed methods study (Ross et al. 2013) [60]	Reduction in depressive symptoms	Individual telephone counselling: focussing on emotional and informational support	N = 40	Individual	Weekly for 15 to 30 min	Compared to CG the IG reduced depressive symptoms after 1 (p = .044) and 2 months (p = .001) of receiving this intervention
Efficacy and cost-effectiveness of drug and psychological treatments for common mental disorders in general health care in Goa, India: a randomised, controlled trial (Patel et al. 2003) [54]	Psychiatric morbidity	A) Antidepressant or Psychological Intervention: key features of which were: 1) explanation of the treatment and reassurance, 2) relaxation (breathing exercises), 3) treatment for specific symptoms (dependent on the patient’s report)—eg, activity scheduling for tiredness or establishing sleep routines for sleep problems, and 4) problem solving; or Placebo	N = 450	Individual	Psychological treatment: weekly intervals for the initial sessions, and fortnightly thereafter for up to six sessions	IG had reduced psychiatric morbidity (measure by CISR total score) than with placebo at 2 months (P = 0.005) but not from 2 to 12 months (P = 0.1). CG was not more effective than placebo for any outcome at all the time points (P = 0.86 at 2 months and P = 0.48 at 2–12 months)
Resource activation for treating post-traumatic stress disorder, co-morbid symptoms and impaired functioning: a randomized controlled trial in Cambodia (Steinert et al. 2017) [66]	Remission rates of PTSD Symptoms of depression and anxiety Self-perception of functioning (SPF) Depression and emotional distress status	ROTATE: Resource-oriented trauma therapy combined with eye movement desensitization and reprocessing (EMDR) resource installation	N = 86	Individual	5 h in total delivered on a weekly basis	IG reported significantly reduced levels of depression, anxiety and impaired functioning and increased PTSD remission rates (P < 0.001) compared with CG
The Effectiveness of Eye Movement Desensitization and Reprocessing Therapy to Treat Symptoms Following Trauma in Timor Leste. (Schubert et al. 2016) [69]	Severity of PTSD, Depression and Anxiety symptoms: Psychophysiological response	EMDR therapy	N = 21	Individual	Up to 10 sessions lasting 60 – 90 min	IG versus CG control was significant in reducing depression, anxiety and PTSD post intervention (P < 0.001), and depression three months later (P = 0.034). There were no significant differences in anxiety or PTSD symptoms 3 months later
Unanticipated effect of a randomized peer network intervention on depressive symptoms among young methamphetamine users in Thailand (German et al. 2012) [57]	Sexual risk behaviour Methamphetamine use Depression	Peer-educator network-oriented intervention	N = 983	Group	Delivered twice weekly, was 7 sessions in duration, with each session lasting 2 h, over the course of a month	Post-assessment the IG group showed a significantly different decreasing trend in depressive symptoms as indicated by the condition-by-time interaction (P < 0.0001) Symptoms scores of PTSD and anxiety were remained the same at 3-month follow-up, but depression scores continued to decrease
Effect of mobile phone-based psychotherapy in suicide prevention: a randomized controlled trial in Sri Lanka (Marasinghe et al. 2012) [70]	Suicidal ideation Depression	Brief Mobile Treatment	N = 68	Phase 1: Group Phase 2: Individual	Phase 1: training in problem solving, meditation, increasing social support skills, as well as advice on alcohol and drug use Phase 2: followed up by 10 phone calls over 24 weeks, along with weekly SMS reminders and 5 min audio voice messages to reinforce the learning from this training	IG reported reduced suicidal ideation and depression in people who recently attempted suicide versus CG at 6 and 12 months (No P-Values reported)
Testimony Therapy With Ritual: a Pilot Randomized Controlled Trial (Esala & Taing, 2017) [67]	Severity of PTSD, anxiety, and depression	Testimony Therapy plus ceremony (culturally adapted ceremony which involves a Buddhist ceremony and a truth-telling event)	N = 120	Both	Included 4 days of individual counselling and a culturally adapted group ceremony on the 5^th^ and final day	Compared to the CG the IG reported significant reduction in depression symptoms p = .001, but symptoms did not significantly decrease from 3 to 6 months. Again, there was a significant reduction in anxiety symptoms for IG from baseline to 3 months, p = .001, but symptoms did not significantly decrease from 3 to 6 months

### Assessing studies based on quality and risk of bias

The full data extraction table and the risk of bias assessment using the Cochrane Risk of Bias tool are presented as Additional files 1, 2. When appraising the quality of the retained studies, there was a large degree of variation in samples sizes, with a minimum of 21 and a maximum of 2796 participants. Sixteen of the thirty two studies had small samples (n < 100), with the total number of participants being 11,143 aged between 18 and 74. The majority of the studies comprised of more women than men and only 4 out of the 32 studies utilised an active control group comparator. The risk of bias was low to high across the studies, but the source of bias varied. The blinding of participants and personnel was deemed to be at a high risk of bias in the majority of studies (n = 19). There was a moderate risk of bias in this domain in 9 studies and low risk in only 4 studies. The blinding of outcome assessment was either high risk, moderate risk or unclear in the majority of studies (n = 22). There was a low risk of bias in this domain for 10 studies. There was low risk of bias in sequence generation in the majority of the studies (n = 22) studies. The risk of bias in allocation concealment in the majority of the studies (n = 19) was either unclear, moderate or high. This means that foreseeable potential selection biases were not ameliorated in the majority of the reviewed studies. The potential risk of bias related to incomplete outcome data and selective outcomes was low in the majority of studies, (n = 18) and (n = 18) respectively. However, a number of studies were subject to moderate to high levels of bias in incomplete outcome data (n = 14) and selective outcomes (n = 11), meaning that overall as a whole this set of studies were deemed to demonstrate a low to moderate risk of bias in these domains. The studies reviewed used a range of validated questionnaires to assess the impact of the interventions on the same outcomes e.g. depression (such as Hamilton Depression Rating Scale (HAM-D), Patient Health Questionnaire (PHQ-9), Beck Depression Inventory-II (BDI-II)) which further hinders direct comparison between studies.

### Effects of interventions or programmes on mental health issues and disorders

The reviewed studies reported a range of outcomes and intervention or programme types. The most often reported outcome in the included studies was depression, which was reported as the primary or secondary outcome in the vast majority of the reviewed papers (n = 25). This was followed by anxiety (n = 10), Post Traumatic Stress Disorder (PTSD) (n = 4), suicidal thoughts or ideation (n = 3), quality of life (n = 3), psychological distress (n = 2), well-being (n = 3), psychiatric morbidity symptoms (n = 2), sleep quality (n = 1) and levels of mindfulness (n = 1). Only two studies reported a cost-effectiveness analysis [49, 54]. The types of intervention or programmes employed were quite disparate in terms of delivery and patient/client groups. A number of programmes used hybrid approaches with a range of modalities included within a programme e.g. counselling and ceremonial rituals, while others adhered more rigidly to their intervention/programme protocols e.g. EMDR or CBT. The intervention programmes reviewed can be categorised as: lay delivered (n = 7) [40, 41, 49, 56, 64, 68, 71], yoga, aerobic and/or meditation based (n = 9) [42–46, 50–52, 58], CBT oriented (n = 8) [47, 48, 53, 59, 61–63, 65] health worker delivered (n = 3) [54, 55, 60]. EMDR oriented (n = 2) [66, 69] and professional hybrid interventions or programmes (n = 3) [57, 67, 70].

### Targets: depression, anxiety, psychological distress, suicidal thoughts

#### Lay delivered interventions

The Thinking Health programme (THP) is a peer delivered programme for perinatal depression which was evaluated by Fuhr et al. [41] and Vanobberghen et al. [71]. THP is based on: behavioural activation, active listening, collaboration with the patient’s family, guided discovery and homework. THP can be delivered individually or in a group format over 6–14 weeks over a 6 month period. In a study in India, Fuhr et al. [41] found that THP intervention plus enhanced usual care versus enhanced usual care significantly improved depressive symptoms (P = 0.01) at 6 months, however rates of remission (P = 0.16) were not significantly different between the groups at 6 months. Vanobberghen et al. [71] pooled the results from Fuhr et al. [41] with another RCT from Pakistan (N = 530) and found that THP was effective at reducing levels of depression (P = 0.03) and remission from depression (P = 0.04) at 6 months. The main difference between the Pakistan versus India delivery was that in Pakistan the intervention was delivered in a mixture of group and individual sessions.

The Healthy Activity Programme (HAP) is a brief psychological treatment adapted from behavioural activation including: psychoeducation, behavioural assessment, activity monitoring, activity structuring and scheduling, activation of social networks, and problem solving. HAP is typically delivered over a 6–8 week period, and sessions can be delivered weekly at home or over the telephone, individually for 30–40 min. In a study of moderately severe to severely depressed primary care patients, Patel et al. [49] (N = 493) found that HAP and enhanced usual care (EUC) versus EUC reduced depressive symptom severity (P < 0.001) and higher remission in depression (P < 0.001) than the control group after 3 months. HAP and EUC also significantly reduced suicidal thoughts or attempts (P < 0.0001). Jordans et al. [64] (N = 120) supported these findings in Nepal, reporting that HAP and EUC versus EUC significantly lower depressive symptom severity (P < 0.001) at 3 and 12 month follow up (P < 0.001) amongst depressed patients in primary care.

MANAS is a collaborative stepped-care intervention which includes case management along with psychoeducation and individual counselling (a minimum of 6 sessions with maximum of 12 sessions) provided by a trained lay health counsellor, which were supplemented by antidepressant medication dispensed by a primary care physician and supervised by a mental health specialist. All interventions are delivered in MANAS at an individual level within clusters over a 6 month period. Patel et al. [56] conducted a large scale cluster randomised controlled trial of MANAS versus enhanced usual care in 12 private and 12 public primary health care centres (N = 2796) in India and Pakistan. Patel et al. [56] found that the intervention group experienced a small significant effect of increased recovery from common mental health disorders (i.e. a co-morbidity of anxiety and depressive disorder measured by ICD-10 criteria) at 6 months (P = 0.02) versus the control group. The control group received the mental health screening results and a treatment manual prepared by the primary care physicians. Patel et al. [56] also conducted a subgroup analysis on participants with depression and no significant difference was found between the intervention and control group (P = 0.07). Patel et al. [40] reported the follow up results of this study after 12 months. They found that those receiving the intervention in public facilities had a 30% reduction in prevalence in ICD-10 diagnosis group (for anxiety and depressive disorders) (P = 0.02) and a 24% reduction in the ICD-10 depression subgroup (P = 0.04). No significant effects were found in either group in the private facilities (P = 0.34 and P = 0.31 respectively). The significant effects being found in public care settings rather than the private care settings was identified by Patel et al. [40] as being potentially due to the fact that the primary care physicians were a highly motivated group of practitioners, who were committed to delivering personalized client-centred care. Patel et al. [40] outlined that this may have cancelled out the additional value of the lay health counsellor in the intervention. 

Sherman et al. [72] developed a peer educator, network-oriented intervention with the goal of reducing methamphetamine use, sexual risks, and incident STIs among young adults in northern Thailand. The intervention provided information about the sexual risks associated with methamphetamine use along with peer education skills building exercises in order to enhance the participant’s capacity to disseminate this information within social and peer networks in order to promote risk reduction norms. This intervention was delivered in groups of 8–12 participants. The intervention was delivered twice weekly, was 7 sessions in duration, with each session lasting 2 h, over the course of a month. German et al. [57] (N = 983) found that this intervention, unexpectedly, decreased depression severity versus a group‐based life skills intervention of the same frequency and duration (P < 0.0001) among young methamphetamine users in Thailand 12 months from baseline.

Markkula et al. [65] developed a counselling intervention, delivered by non-medial health workers, which focussed on providing emotional support along with the development of problem solving and coping skills, in order to reduce psychological distress in a primary care setting in rural Nepal. This intervention, which was delivered individually, consisted of five 45 min appointments, two of which were completed in the first week, with meetings being weekly from weeks two to four. Markkula et al. [65] (N = 287) found that this intervention was effective versus enhanced usual care at improving depressive and anxiety symptoms at 1 and 6 months (P-Values not reported).

Arjadi et al. [68] developed an internet-based behavioural activation programme, supported by lay counsellors, to reduce depression in adults who met the criteria for depressive disorder within a community setting in Indonesia. The programme consisted of a series of eight weekly structured modules, with assignments, that could be completed individually in 30–45 min. Each module focused on developing the participant’s capacity to monitor their daily mood and encouraged participants to complete pleasurable, mood-independent, pre-planned activities. Lay counsellors provided additional support if needed, by giving feedback on assignments (which took approximately 30–60 min a week) and by providing reminders to participants to engage with the programme regularly. Arjadi et al. [68] (N = 314) found that this intervention led to significantly lower depression scores versus online psychoeducation (P = 0.017). These results were maintained at 3 and 6 months.

### Targets: depression, anxiety, wellbeing

#### Yoga, aerobic and/or meditation based interventions or programmes

There were a number of studies which identified the effectiveness of yoga interventions solely, or yoga mixed with meditation. Tolahunase et al. [42] examined the effectiveness of a yoga and meditation lifestyle intervention (YMLI) on major depressive disorder (MDD) in India. This intervention incorporated physical yoga poses, breathing exercises and meditation. The programme consisted of sessions lasting for 2 h, practiced for 5 days a week over 12 weeks. The first 2 weeks of the intervention included supervised group sessions, which included lectures on yoga, meditation, major depressive disorder and the importance of depressive symptom prevention and management. The remaining 10 weeks was completed individually at home, including one to one and unsupervised sessions. Tolahunase et al. [51] (N = 178) found that YMLI had a significant effect on depression severity versus drug treatment (P < 0.001). A stratified analysis found that this change was significant for those deemed to have moderate MDD (P = 0.029) and severe MDD (P < 0.01) at baseline but not mild MDD (P < 0.072). Tolahunase et al. [51] found that YMLI significantly improved depression severity in people with depressive disorder who were on drug treatment for at least 6 months versus TAU (P < 0.001).

Yoga Nidra is a deep relaxation technique [44]. Rani et al. [44] developed a Yoga Nidra intervention protocol to improve the wellbeing and reduce depressive and anxiety symptoms in women with menstrual irregularities. This protocol consisted of Yoga Nidra classes being delivered in groups, for 35 min a day, 5 days a week for 6 months. Rani et al. [44] (N = 150) found that Yoga Nidra versus TAU improved depressive symptoms for women with menstrual irregularities (P < 0.01) and anxiety (P < 0.01) after 6 months. Rani et al. [52] (N = 150) using the same data set but reporting the use of HAM-D for depression and HAM-A for anxiety found that Yoga Nidra improved mild to moderate depression and anxiety symptoms of women with menstrual disorder HAM-A (mild P < 0.002, moderate P < 0.03) and HAM-D (mild P < 0.02, moderate P < 0.05) versus the control group after 6 months but not severe depressive symptoms. In a separate RCT, Rani et al. [45] (N = 100) found that Yoga Nidra therapy and pharmacotherapy improved depressive (P < 0.02) and anxiety symptoms (P = 0.003) and positive well-being (P = 0.02) of women with menstrual disorder after 6 months versus pharmacotherapy.

Chan et al. [73] developed an integrated body-mind-spirit group intervention (IBMSGI). The intervention includes lectures on emotional and health management, stress reduction training (including breathing and meditative techniques), acupressure exercises, and homework activities such as writing and drawing, which encourage participants to focus on positive meanings within negative experiences [50]. This intervention is delivered in small group sessions, lasting 3 h in duration, once per week for 4 weeks [43] Rentala et al. [43] (N = 30) found that IBMSGI versus TAU (antidepressants, structured psychoeducation and brief counselling) was effective at reducing depression among depressive patients over a 3 month period (P < 0.001). Rentala et al. [50] (N = 120) in a larger scale RCT of IBMSGI and the same control found that IBMSGI versus TAU was effective at reducing depression among depressive patients over a 6 month period (P < 0.001) and improving well-being (P < 0.001) and quality of life (P < 0.001).

Roy et al. [46] with the guidance of experts in the field of mental health and aerobic experts developed the Video Assisted Structured Aerobic Exercise Program (VASAEP) to improve mood and somatic symptoms among women with depressive disorders. The VASAEP consists of 20 min of moderate to low intensity aerobic exercises, delivered in a group format via a laptop, supervised by a registered nurse, every day for 10 days. Roy et al. [46] found that VASAEP versus TAU improved levels of depression (P < 0.05) at the programme’s end but not mood score or somatic symptoms related to depression.

Siritienthong et al. [58] examined the effectiveness of a mindfulness based group therapy intervention (not identified as either Mindfulness-based Stress Reduction or Mindfulness-based Cognitive Therapy) on sleep quality and mental health symptoms of people with chronic insomnia in Thailand. This mindfulness based group met weekly for 1.5–2 h for 8 weeks, though the authors provide very limited information on the interventions weekly content. Siritienthong et al. [58] (N = 20) found no differences between this intervention and TAU on sleep quality (P = 0.76), depression symptoms (P = 0.49), anxiety symptoms (P = 0.14) or mindfulness levels (P = 0.25). This may however be due to the fact that the ‘quasi randomisation’ procedure engaged in this study appears to have failed, as both groups appear to have significantly different scores on each variable at baseline. No tests to examine systematic differences on each variable at baseline were employed in this study. The intervention group also had low levels of anxiety and depression on the HADS-A and HADS-D, meaning that a floor effect could have impacted the results.

### Targets: depression, psychological distress, quality of life, PTSD, complicated grief

#### CBT oriented interventions or programmes

There were a number of small scale RCT studies which focussed on the use of CBT only, behavioural activation or CBT with psychoeducation and relaxation techniques. Thapinta et al. [48] (N = 80) found that 6 sessions of group CBT delivered over a 3 week period was effective at reducing depression among Thai general hospital patients with alcohol dependence post intervention (P < 0.01) and 7 weeks later (P < 0.01) versus usual care. In an RCT of a 6 week group CBT intervention (versus a control—type not reported), which included psychoeducation and relaxation exercises for depression among menopausal women, Reddy et al. [47] (N = 102) found that the CBT intervention significantly improved depression symptoms (P = 0.00) over a 6 month period. Indu et al. [53] developed an 8 week CBT oriented intervention including psychoeducation and behavioural activation which could be delivered individually by health workers. This intervention was then combined with drug treatment to help manage depression in women in a primary care setting in India. Indu et al. [53] (N = 60) found that this integrated intervention had a large effect on depressive severity (P = 0.01) and quality of life (P = 0.006) at 8 weeks versus referral to available services as TAU. Bryant et al. [59] (N = 28) found that 8 sessions of CBT for terrorist attack survivors, delivered individually, significantly improved PTSD (P = 0.001), depressive symptoms (P = 0.004) and complex grief post treatment symptoms (P = 0.001) post intervention and 3 months (P = 0.007 for PTSD; P = 0.003 for depression and P = 0.003 for complicated grief) post treatment versus TAU. Songprakun and McCann [61–63] (N = 56) found that an 8 week self-help and CBT based manual for depression plus standard care and treatment versus standard care and treatment improved resilience levels (P = 0.029), and depression post treatment (P = 0.018) and 4 weeks hence (resilience P = 0.004; depression P = 0.005) but not psychological distress. The lack of a finding for psychological distress may be due to the different group scores at baseline.

### Targets: depression, schizophrenia

#### Health worker delivered interventions or programmes

Balaji et al. [74] developed the Community care for people with Schizophrenia in India (COPSI) programme. This programme includes structured needs assessment, individualised treatment plans, structured multidisciplinary team clinical reviews and supervision from community health workers. The programme also included psychoeducation for participants and caregivers, medication adherence support, physical health promotion, rehabilitation strategies to improve personal, social and work functioning, supports to deal with stigma and discrimination, self-help groups and linking in with other networks of community support to address social issues, social isolation, access to legal benefits and employment opportunities. Chatterjee et al. [55] (N = 282) found that COPSI delivered by community health workers in the intensive engagement (0–3 months), stabilization (4–7 months), maintenance phases (8–12 months) versus facility-based care (TAU) reduced positive and negative symptoms of schizophrenia versus usual care at 12 months (P = 0.01). Ross et al. [60] in a study of depression rates among HIV-Infected pregnant women in Thailand (N = 40) found that individual telephone counselling support from a registered nurse reduced depressive symptoms after 1 (P = 0.04) and 2 months (P = 0.001) of receiving this intervention versus usual care.

### Targets: depression, anxiety and PTSD

#### EMDR based interventions or programmes

In an RCT of an EMDR intervention to treat trauma symptoms in post war Timor Leste, Schubert et al. [69] found that EMDR – delivered individually, for up to 10 sessions, lasting 60–90 min in duration (N = 21) versus waitlist control was significant in reducing depression, anxiety and PTSD post intervention (P < 0.001), and depression 3 months later (P = 0.034). There were no significant differences in anxiety or PTSD symptoms 3 months later. Wöller and Mattheß [75] developed ROTATE, a trauma therapy which focussed on the development of a secure therapeutic relationship and enhancing patient resilience and coping capabilities through the use of EMDR techniques. Steinert et al. [66] (N = 86) found that Cambodian patients with PTSD, who received 5 h of ROTATE, delivered individually on a weekly basis, had significantly reduced levels of depression, anxiety and impaired functioning and increased PTSD remission rates (P < 0.001) versus a waitlist control group.

### Targets: depression, anxiety, PTSD, suicidal ideation, self-harm, psychiatric morbidity

#### Professional hybrid interventions or programmes

We found three other RCT studies which focussed on a hybrid form of psychological and social intervention, with a mix of modalities. In an RCT of testimony therapy which included 4 days of individual counselling and a culturally adapted group ceremony on the 5th and final day versus waitlist control, Esala and Taing [67] found that the intervention group’s PTSD (P < 0.001), anxiety (P < 0.018) and depression (P < 0.003) improved 3 months post intervention but not at 6 months.

Marasinghe et al. [70] developed Brief Mobile Treatment (BMT) in order to reduce suicidal ideation and self-harm in people who attempted suicide in Sri Lanka. The treatment includes individual mental health assessment and then allocation to a group which receives training in problem solving, meditation, increasing social support skills, as well as advice on alcohol and drug use. This was then followed up by 10 phone calls over 24 weeks, along with weekly SMS reminders and 5 min audio voice messages to reinforce the learning from this training. Marasinghe et al. [70] (N = 68) found that BMT reduced suicidal ideation and depression in people who recently attempted suicide versus those receiving usual care at 6 and 12 months (No P-Values reported). No significant effect on reducing self-harm was found at 6 or 12 months in this study.

Patel et al. [54] (N = 450) conducted the only randomised double blind placebo controlled trial of this review which tested the effectiveness of (1) antidepressants (fluoxetine), (2) a placebo (both administered for 6 months) or (3) psychological treatment (6 individual session over a 3 month period) by a trained therapist for adult out-patients who attended for treatment at the district general hospitals. Psychological treatment included: explanation of the treatment, reassurance, relaxation exercises, treatment for specific symptoms (depending on the patient’s needs e.g. sleep routines) and problem solving. Patel et al. [54] found that antidepressants were better at reducing psychiatric morbidity (measure by CISR total score) than with placebo at 2 months (P = 0.005) but not from 2 to 12 months (P = 0.1). Psychological treatment was not more effective than placebo for any outcome at all the time points (P = 0.86 at 2 months and P = 0.48 at 2–12 months).

## Discussion

This systematic review has identified and systematically analysed the available RCT evidence on the effectiveness of psychological and social interventions or programmes to support people with mental health issues or disorders in Southeast Asia. The reviewed studies reported a range of outcomes and intervention or programme types. The heterogeneity across the studies in terms of programme content, delivery, duration, and study sample makes it difficult to draw general conclusions about the effectiveness of these interventions as a whole, so this discussion will focus on the strength and consistency of evidence for each intervention type. To date, to the best of our knowledge, no other systematic review that addresses the effectiveness of a range of psychological and social mental health interventions or programmes for supporting people with mental health issues or disorders in Southeast Asia exists. Even though this review’s intervention type and outcome inclusion criteria were wide, the review only contained 32 RCTs, which examined the effectiveness of a broad range of interventions on a broad range of mental health issues and disorders. A recent systematic review of RCTs which had a narrower focus examining the effectiveness of only one intervention type and outcome covered in this RCT (CBT for depression in primary care) by Santoft et al. [76] found 34 papers met their inclusion criteria. The majority of the research conducted in this review was in India (16 out of 32) a country of 1.4 billion people [77] and combined prevalence estimate of over 95 million people with depressive or anxiety disorders [3]. There was only one study conducted each in Pakistan (whose results were pooled with an Indian RCT), Indonesia and Timor-Leste, countries with a combined population of 497.5 million people [77] and a combined prevalence of 26.5 million people with either a depressive or anxiety disorder [3, 77]. The majority of the RCTs that did exist were small or medium in size. There was also only two RCTs which reported a cost effectiveness analysis. The limited number of RCTs perhaps reflects the fact that mental health tends to be a very low priority for government spending in Southeast Asia [4, 8, 9, 11, 13], and thus each government’s prioritisation of research in this area has suffered as a result [19]. It is encouraging to note that there has been a significant increase in RCT publications in the region since 2015, with 18 out of the 32 RCTs being published since then. There does appear however to be a chronic shortage of quality RCT research studies being conducted on mental health interventions or programmes in the region. This shortage means that a clear gap is present between research, policy-making and practice which is likely to reduce Southeast Asian government’s capacity to take the deliberate, concrete and targeted steps necessary to address the complex mental health needs of their populations [11, 22].

While trained professionals are essential to provide specialised services, one strategy to address human resource shortages is to provide tailored training to professionals from other sectors or non-professionals e.g. peers or lay people, and allow them to administer psychological and social mental health interventions [78, 79]. This process is referred to as task shifting and the highest quality and most promising findings from this review suggest that this approach may be effective in Southeast Asia [79]. The RCTs of these psychological and social interventions were a consistently higher standard than the literature reviewed on other interventions e.g. yoga or CBT oriented, which had much lower sample sizes and higher risks of biases. In India, Patel et al. [40] over 12 months, found that a collaborative stepped care intervention resulted in significantly faster recovery rates for anxiety and depressive disorders in public health care patients. THP [41, 71] and HAP [49], two promising peer led interventions were both found to reduce levels of depression and depressive remission in India and Pakistan, with HAP also reducing suicidal thoughts or attempts over 12 months in a medium sized RCT in Nepal [64]. A group based peer led life skills intervention [57], a lay counselling intervention [65] and an online behavioural activation programme with peer support [25] were also found to reduce depressive and/or anxiety symptoms at 3, 6 and 12 months. These results are in line with the effectiveness of task shifted psychological and social interventions in other low income countries e.g. Spedding et al. [79] found preliminary support for the adaptation of manualised, evidence-based task shifted programmes in public mental health in South Africa in the treatment of depression. The results from these interventions are promising, particularly as they appear to be effective across a range of important mental health outcomes e.g. depression and anxiety disorder, which are the leading causes of years lived with a disability in LMICs [5]. These types of intervention programmes may also have additional benefits, not assessed in this review, such as: earlier identification of mental health issues through enhanced screening mechanisms, enhanced community engagement with, access and uptake of services, increased capacity of remitted patients to engage in work or educational opportunities, stigma reduction and better treatment adherence [80]. Further replication and long-term evaluation studies are needed in different countries to evaluate the generalisability of these interventions across the wider Southeast Asian context [81]. The studies reviewed however do demonstrate the feasibility and potential sustainability of implementing task shifted interventions in Southeast Asia through employing existing human resources and infrastructures, and/or employing peers or lay health workers to deliver interventions [81]. There is however a lack of research in the Southeast Asian context on the effectiveness of these interventions on other important mental health outcomes e.g. mental wellbeing [82]. Future research should begin to fill this research gap through the implementation of high quality RCT research designs with active comparison control groups. This research will likely lead to a wider range of enhanced mental health outcomes and increased access to mental health services for underserved populations in Southeast Asia [83].

Recognition of the common goal that Buddhism, medicine and psychology each have in reducing suffering has helped to pave the way for the entry of yoga and meditative exercises into mental health programmes in higher income countries [84]. Yoga and meditative practices are at the heart of ancient Buddhist and Hindu traditions, and as such have been practiced, analysed, and debated for in Southeast Asian countries such as India, Nepal, Sri-Lanka, Thailand, Bhutan and Cambodia [85, 86]. This review however contains a very modest number of small to medium scale RCTs on the use of yoga only or along with meditative practices as an intervention or treatment programme for mental health, with one very small scale RCT, with significant design issues [58] investigating an 8 week mindfulness based therapy. The limited amount of literature which has been produced on the effectiveness of such interventions in Southeast Asia again highlights the gap between the volume and extent of RCT research activity being conducted in other parts of the world. A recent systematic review, which evaluated the effectiveness of yoga on depressive symptoms in people with mental disorders [87] reviewed 19 RCTs. The limited amount of literature available that we reviewed does provide some promising preliminary evidence that yoga and/or meditation could reduce the prevalence of depressive disorders, symptoms of depression and anxiety and improve well-being in both depressive patients [50, 51] and in women with menstrual disorder in Southeast Asia [52]. These results are in line with Cramer et al. [88] who in a systematic review of 12 RCTs which examined the effectiveness of mind–body yoga and relaxation interventions found that such interventions should be considered as a treatment option for patients with depressive disorders and individuals with elevated levels of depression. In order to attain more convincing evidence of the feasibility and effectiveness of these interventions in a Southeast Asian context however, much more research of a higher quality is needed e.g. future RCTs should contain larger sample sizes and the use of active control groups. The feasibility of the implementation of yoga-mindfulness-based interventions in supporting mental health outcomes in LMICs in Southeast Asia is encouraging. Since the 2000s, Mindfulness Based Stress Reduction (MBSR) and later Mindfulness-based Cognitive Therapy (MBCT) (secular interventions derived from ancient Buddhist meditative teachings) [89] have seen an exponential research growth trajectory in the fields of psychology, psychiatry, medicine and neuroscience in higher income countries [90]. The growing importance of these interventions, is in part, fueled by the empirically supported, clinical effectiveness of MBIs in dealing with a large variety of acute and chronic physical and psychological disorders [86, 91–93]. Systematic reviews and meta-analyses have found that MBIs have positive effects on mental health issues such as anxiety, depression, PTSD, psychiatric conditions and mental wellbeing with a range of clinical samples across age groups [86, 94–100]. MBIs are also relatively brief, empowering [101] and cost effective programmes [102] that can potentially support a range of mental health issues, and co-morbid symptomologies. Given the high levels of prevalence of mental health issues and disorders in Southeast Asia, and the limited clinical armamentarium available to meet the mental health needs of Southeast Asian populations, research into the effectiveness of MBIs in a Southeast Asian context should be undertaken, again through high quality RCT methodological designs.

There was also a number of small-scale RCT studies which evaluated the effectiveness of a range of interventions with individual and multiple components. These included CBT only or CBT with psychoeducation and relaxation techniques, EMDR or hybridised interventions. This literature covered a wider range of patient groups and populations and the results from these studies were less conclusive. This literature provided some initial preliminary evidence that CBT may be effective at reducing depression among patients with alcohol dependence, menopausal women and terrorist attack survivors (along with PTSD in this group) [47, 48, 53, 59, 61, 63, 65]. The RCTs reviewed also provide some initial preliminary evidence that CBT may be effective at reducing depression along with improving resilience and quality of life in people with depression. EMDR focussed or adapted interventions with no active control group were found to reduce depression, anxiety and depression in patients who had experienced PTSD in Cambodia [66] and Timor Leste [69] post intervention, but not 3 months later in the Timor Leste study. This preliminary evidence highlights the potential of these more westernised interventions in meeting the mental health needs of Southeast Asian populations. A number of systematic reviews have highlighted CBT’s effectiveness at improving a number of mental health outcomes e.g. PTSD [103] anxiety in older people [104] and depression in primary care [76] EMDR also has an established evidence base in reducing symptoms and improving remission rates in adults with PTSD, even in the long term [105]. Other preliminary evidence also suggests that EMDR may also be an effective treatment for both anxiety [106, 107] and depressive disorders [108, 109]. Future research in Southeast Asia should examine the effectiveness of both of these interventions through high quality RCT designs. The identification of whether CBT, EMBR, MBIs and/or task shifted interventions are effective, will help to ensure that limited financial resources allocated to mental health services in these countries are spent more efficiently [4, 8–11].

In Cambodia, testimony therapy was found to improve PTSD, anxiety and depression symptoms 3 months post intervention but not at 6 months. A Brief Mobile Treatment intervention [70] reduced suicidal ideation and depression but not self-harm in people who recently attempted suicide versus those receiving usual care at 6 and 12 months. This literature suffers from even more methodological issues than the yoga and meditation, CBT and EMDR RCT literature discussed earlier e.g. higher risks of bias and smaller sample sizes. It is thus clear, that to be more convinced about the effectiveness of these, and the other intervention or programme types reviewed in this paper, in a Southeast Asian context, that significantly more research, of a higher methodological quality is needed. This is perhaps evidenced by the fact that the only randomised double blind placebo controlled trial in this review by Patel et al. [54] found that psychological treatment, which included: explanation of the treatment, relaxation exercises, treatment for specific symptoms (depending on the patient or client’s needs e.g. sleep routines) and problem solving, was not more effective than a placebo at improving common mental health disorders at 2 or 12 months.

Historically, the limited number of intervention and RCT studies that have been conducted in Southeast Asia have had small samples sizes, along with heterogeneity in participant recruitment mechanisms, methodological design and outcome measurement [110]. This still appears to be the case. The majority of the studies included in this review were of low to moderate quality due to the variability in the quality of the study design, the small samples sizes recruited and short term duration of the evaluations [36, 37]. The majority of the studies reviewed also did not contain an active control comparison group. This means that in these studies, we cannot exclude that the differences between the intervention and control groups were not simply due to non-intervention related factors e.g. receiving attention, expecting to improve due to receiving an intervention or having a pleasant social experience [111]. The limited nature of the assessment and reporting of treatment fidelity in the reviewed studies also limits the reliability and validity of their results [112, 113]. The external validity of the reviewed studies is also limited by self-selection bias. This is due to the fact that participants were randomly assigned from a motivated group of participants who wanted to be part of the study. This makes it difficult to establish how representative these groups were on the general populations in the respective countries in which the studies took place [111]. The blinding of participants, personnel or outcome assessor bias was either high risk, moderate risk or unclear in the majority of studies. However, due to the nature of the interventions evaluated, it would not have been possible to blind the participants to treatment conditions in the majority of these studies e.g. testimony therapy versus waitlist control [67]. As a result of these biases however, performance or detection biases could have affected the self-report outcome measures [36, 37]. The studies reviewed also used a range of self-report measures, thus common methods bias, which could have inflated the effects of the interventions in the reviewed studies [114]. The wide range of measures used to measure the same outcome e.g. depression, further hindered comparison between studies. The majority of the measures used in the reviewed literature were existing standardized mental health measures from higher income countries with the reviewed studies engaging in limited exploration of how culturally valid these were. In order for future RCTs to more accurately evaluate interventions in diverse cultural contexts, the cultural validation of existing measures, or the development of culturally valid measures and sensitive indicators of mental health issues and disorders is likely to be needed [81]. In order to increase the quality of the research examining the effectiveness of psychological and social interventions reviewed in Southeast Asia going forward, future RCT research should ameliorate bias issues where possible e.g. through the use of active control group comparisons and greater measurement of treatment fidelity.

A key element of evidence-based treatment in psychological and social interventions is finding out which forms and types of therapy work for which individuals under what circumstances [115]. The effectiveness of the interventions reviewed may entail multiple mechanisms that vary with different clinical populations. Thus, more work is needed to ascertain which variations in which mechanisms are most effective for which groups under what circumstances [116]. Understanding how and why the reviewed interventions can effectively improve the mental health outcomes of people from Southeast Asian countries is essential both for theoretical and clinical reasons [117–119]. By identifying the mechanisms of action in these interventions and enhancing the theoretical understanding of how these treatments may work, therapeutic effects can be optimized through: the enhancement of the active components of interventions (enhancing efficacy through these mechanisms), the identification of treatment mediators and moderators, which will improve clinician's ability to match patient or client groups to potentially beneficial interventions or programmes [117–119]. Studying the mechanisms of therapeutic change in these interventions might reveal any shared variance between potential mediators and contribute to a better understanding of possible causal relationships in the processes that mediate between these interventions and mental health outcomes [117, 119]. For example, in the case of depression as an outcome, some of the components of specific interventions may influence depression only indirectly, through their influence on other variables such as worry or rumination, while others may exert both direct and indirect influences on depression [120, 121]. Thus, more studies are required to measure training-related change mechanisms, their relationships to one another, and how these relationships achieve a therapeutic benefit for intervention participants [120, 121].

## Limitations

This systematic review provides a useful synthesis of the current evidence regarding the effectiveness of psychological and social interventions for adults with mental health issues or disorders in Southeast Asia. This review does however have a number of limitations. Studies not employing RCT designs were excluded from the search and therefore, qualitative and other potential intervention evaluation study designs were discarded in the search process. Second, the broad inclusion criteria which led to the inclusion of heterogeneous psychological and social interventions and outcomes meant that a meta-analysis could not be conducted. Despite these limitations, the studies included in this review clearly demonstrate that high quality and effective psychological and social mental health interventions, and their evaluation through well-designed research studies, are feasible in Southeast Asian countries.

## Conclusions

The review findings indicate that a range of mental health interventions can be implemented effectively in Southeast Asian mental health and health settings. There is reasonable quality and promising evidence that multicomponent task shifted lay and peer led interventions may be effective at improving a range of mental health outcomes in Southeast Asia. These interventions potentially allow the wider accessibility of mental health interventions or supports to larger numbers of people in resource restricted countries in Southeast Asia [81]. Other intervention types, which rely on trained professionals e.g. yoga, meditation, CBT or EMDR also show promise. On balance, much more research, through higher quality and larger scale RCTs, funded through greater Southeast Asian government funding allocations and international funding bodies, will be needed before it can be known definitively for what mental health issues and disorders, in which individuals, these interventions are helpful in Southeast Asia [122]. The evidence that would accrue from such research would likely close the gap between research, policy-making and practice. This research would increase the capacity of Southeast Asian governments to develop and/or implement evidence-based health policies while improving professional practice by making it more evidence informed [19, 23, 24]. This would likely lead to the more effective and more comprehensive addressing of the needs of people with mental health issues and disorders in their countries [3, 22–24].

## Supplementary Information

**Additional file 1. **Data extraction table.

**Additional file 2. **Cochrane Risk of Bias Tool for RCTs.

## Data Availability

Not applicable.
