# Supplementary material for: Psychological and social interventions for mental health issues and disorders in Southeast Asia: a systematic review
Source: Int J Ment Health Syst. 2021 Jun 5;15:56. doi: 10.1186/s13033-021-00482-y (PMC8178881; doi:10.1186/s13033-021-00482-y)
Supplement: Supplementary file 1 — Additional file 1. Data extraction table. [file 13033_2021_482_MOESM1_ESM.docx]

Additional File 1: Data extraction table

| **Study, Author** | **Study design** | **Participants** | **Intervention** | **Mental Health Issue or Disorder Outcomes Targeted** | **Clinical Scales** | **Country** | **Time-frame** | **Key Findings** |
| --- | --- | --- | --- | --- | --- | --- | --- | --- |
| **The Healthy Activity Program (HAP), a lay counsellor-delivered brief psychological treatment for severe depression, in primary care in India: a randomised controlled trial. (Patel et al., 2017) [49]** | Randomised controlled trial    Active control group - no | Age: 18-65 recruited primary care N= 495  IG (n=247) CG (n=248) | Usual care and HAP vrs TAU | Depression Severity   Remission From Depression   Suicidal Thoughts | BDI-II  WHODAS II  BADS  PHQ-9 | India | Baseline, post intervention, 3 months | IG experienced reduced depressive symptom severity (P<0.001), higher remission in depression (P<0.001) and reduced suicidal thoughts or attempts (P<0.0001) versus CG after 3 months. |
| **Effectiveness of the Thinking Healthy Programme for perinatal depression delivered through peers: Pooled analysis of two randomized controlled trials in India and Pakistan. (Vanobberghen et al., 2020) [71]** | Randomised controlled trial    Active control group - no | Women with perinatal depression - 850 women - 280 in India and 570 in Pakistan  18+ | THPP plus EUC vs EUC-only. | Depression  Symptom Severity Remission | PHQ-9 | India and Pakistan - two RCTS pooled | 3 and 6 months post-childbirth | Compared to CG the IG was effective at reducing levels of depression (P=0.03) and remission from depression (P=0.04) at 6 months. |
| **Effectiveness of an intervention led by lay health counsellors for depressive and anxiety disorders in primary care in Goa, India (MANAS): a cluster randomised controlled trial. (Patel et al., 2010) [56]** | Randomised controlled trial    Active control group - no | Primary care (24 centres in India – 12 public and 12 private - 6 allocated in each to intervention group)  Age = 18+  IG (N=1160) CG (N=1269) | Lay Counsellors Psychoeducation  and TAU vrs TAU | Common Mental Health Disorders  Disability  Suicidal Thoughts | CIS-R  WHODAS II | India | Baseline, six month | This study conducted a sub group analysis on participants with depression and no significant difference was found between the IG and CG (P=0.07). |
| **Lay health worker led intervention for depressive and anxiety disorders in India: impact on clinical and disability outcomes over 12 months. (Patel et al., 2011) [40]** | Randomised controlled trial    Active control group - no | Age: 18+. Public clusters (N=823 in IT - 825 in CT) Private clusters - (N=537 in IT group and 611 in CG). | Lay Counsellors Psychoeducation Primary care (24 centres in India – 12 public and 12 private - 6 allocated in each to intervention group) vrs TAU | Common Mental Health Disorder Disability  Suicidal Thoughts | CIS-R  WHODAS II | India | Baseline, 2, 6 and 12 months | The overall effect was a 30% reduction in prevalence in the ICD-10 diagnosis group between arms (P = 0.02); 24% reduction in the depression subgroup (P = 0.04); 34% reduction in the screen-positive group (P = 0.03); and 57% reduction in the sub-threshold subgroup (P = 0.11). Results not sustained at 6/12 months. |
| **Delivering the Thinking Healthy Programme for perinatal depression through peers: an individually randomised controlled trial in India. (Fuhr et al., 2019) [41]** | Randomised controlled trial    Active control group - no | Woman with perinatal depression  (n=280)  CG:(n=140)  IG: (n=140) | Usual care and HAP vs.  TAU | Depression Severity  Remission From Depression | Patient Health - PHQ-9  BDI-II  WHODAS-II  MSPSS. | India | Baseline, Post, 3 months and Six months | IG reported reduced depression symptom severity at 3 months (P=0.01), disability (P=0.009) relative to the CG. The IG also reported improved recovery (P=0.03). No evidence of an intervention effect on remission at 3 months (P=0.08) or WHO-DAS score at 6 months (P=0.16). |
| **Internet-based behavioural activation with lay counsellor support versus online minimal psychoeducation without support for treatment of depression: a randomised controlled trial in Indonesia (Arjadi et al., 2018) [68]** | Randomised controlled trial Active control - yes | Participants (n=313) with depression  IG (n=159 assigned to GAF-ID group CG (n=154) to online psychoeducation | Online behavioural activation with peer support vrs online psycho-education | Depression  Fear and avoidance  Social Support  Quality of Life | PHQ-9  MSPSS  WHOQOL  SCID-5  IDS-SR | Indonesia | Baseline, 3 months, 6 months | IG delivered by lay counsellors with peer support was led to significantly lower depression scores versus CG (P=0.017). These results were maintained at 3 and 6 months. |
| **Effectiveness of psychological treatments for depression and alcohol use disorder delivered by community-based counsellors: two pragmatic randomised controlled trials within primary healthcare in Nepal (Jordans et al., 2019) [64]** | Randomised controlled trial    Active control group - yes | Eligible for RCT (n = 312) Received diagnosis of depression (n = 137).  IG (n = 60)  CG (n = 60) | HAP (for depression) vrs TAU (psychoeducation and pharmacological treatment) | Depression Substance Use Disorder | PHQ-9 WHODAS | Nepal | Baseline, post intervention, 3 months and 12 months | IG vrs CG reported significantly lower depressive symptom severity (P<0.001) at 3 and 12 month follow up (P<0.001) amongst depressed patients in primary care. |
| **5-HTTLPR and MTHFR 677C>T polymorphisms and response to yoga-based lifestyle intervention in major depressive disorder: A randomized active-controlled trial. (Tolahunase, 2018a) [42]** | Randomised controlled trial    Active control group - yes | 178 Patients with MDD  Age: 20-60 | YBLI (YOGA arm) or routine drug therapy with SSRIs (DRUG arm)  Vrs  Drug therapy: SSRIs were used as per the prescription of treating psychiatrists. | Major Depressive Disorder | BDI-II | India | Baseline and post intervention | The IG had a significant effect on depression severity versus CG (P<0.001). A stratified analysis found that this change was significant for the deemed to have moderate (P=.029) and severe MDD (P<0.01) at baseline but not mild MDD (P<.072) after YMLI compared to CG. Clinical improvement was more significant for the women in IG (P = 0.032) |
| **Comparative Effectiveness of Mindfulness-Based Therapy**  **in Sleep Quality of Chronic Insomnia Compared to**  **Standard Cognitive Behavioral Therapy [CBT-I]:**  **A Randomized Controlled Trial. (Siritienthong et al., 2018) [58]** | Randomised controlled trial  Active control group - yes | 25 Patients diagnosed with insomnia (13 in IG and 12 in CG)  Age: 18+ | Mindfulness Based Therapy Group –versus Standard CBT for insomnia in control | Sleep Quality Depression Anxiety  Mindfulness level | Thai-  PSQI.  Thai – HADS.  Srithanya Sati Scale [SSS]. | Thailand | Baseline and post intervention | There were no differences between the IG and the CG on sleep quality (P=0.76), depression symptoms (P=0.49), anxiety symptoms (P=0.14) and mindfulness levels (P=0.25). |
| **Yoga- And Meditation-Based Lifestyle Intervention Increases Neuroplasticity and Reduces Severity of Major Depressive Disorder: A Randomized Controlled Trial. (Tolahunase et al., 2018b) [51]** | Randomised controlled trial    Active control group - no | 58 MDD patients diagnosed and on drug treatment for at least 6 months  Age: 19-50 | 12-week pre-tested YMLI program modified for MDD patients in the current study Vs. TAU | Major Depressive Disorder | BDI-II | India | Baseline and post intervention | For the IG there was a significant decrease difference between means in BDI-II (depression) score (P < 0.001) and significant increase in BDNF (Brain-derived neurotrophic factor) (P < 0.001) post 12- weeks compared to the CG. |
| **Effectiveness of integrated body-mind-spirit group intervention on the well-being of Indian patients with depression: a pilot study (Rentala et al., 2013) [50]** | Randomised controlled pilot study    Active control group – no | Patients with depression (n=30)  IG (n=15)  CG (n=15) | BMS intervention  vs.  Routine hospital treatment. (antidepressants and structured psycho-education) | Depression  Well-being  Work and social adjustment | BDI-II BMSWBI  WSAS | India | Baseline, 1 month, 2 months, 3 months, 6 months | Compared with the CG group, the IG group showed statistically significant decreases in depression (P < 0.001) and functional impairment (P < 0.001)   and statistically significant increases in the well-being (P < 0.001) over the 6-month interval. |
| **Effectiveness of body-mind-spirit intervention on well-being, functional impairment and quality of life among depressive patients - a randomized controlled trial (Rentala et al., 2015) [43]** | Randomised controlled trial    Active control group - no | Patients with depression (n= 120).  CG (n = 64). IG (n = 56) | BMS intervention  vs.  Routine hospital treatment (antidepressants and structured psycho-education) | Depression  Well-being  Functional impairment Quality of life | BDI-II BMSWBI  WSAS WHO QOL BREF | India | Baseline,  1 month,  2 months  3 months  6 months | Compared with the CG group, the IG group showed statistically significant decreases in depression and functional impairment (P < 0.001) and statistically significant increases in the well-being and quality of life (P < 0.001) over the 6-month interval. |
| **Impact of Yoga Nidra on psychological general well-being in patients with menstrual irregularities: A randomized controlled trials (Rani et al., 2011) [44]** | Randomised controlled trial    Active control group - no | Women with menstrual irregularities (n= 150)  IG (n=75)  CG (n=75) | Yoga Nidra therapy and pharmacotherapy  vs.  Pharmacotherapy | Well-being, Anxiety Depression | PGWBI (Translated Hindi version) | India. | Baseline, 6 months | After six months those in the IG compared with the CG had significant reduction in Anxiety (t-test, P value) 3.00 0.003 Depression 2.57 0.01 and Positive well-being 2.26 0.02. |
| **Yoga Nidra as a complementary treatment of anxiety and depressive symptoms in patients with menstrual disorder. (Rani et al., 2012) [52]** | Randomised controlled trial    Active control group – no | Women with menstrual irregularities (n= 150)  IG (n=75)  CG (n=75) | Yoga Nidra therapy and pharmacotherapy  vs.  Pharmacotherapy | Severity of anxiety and depressive symptoms | HAM-A HRSD, HAM-D | India. | Baseline, 6 months | Compared to the CG the IG improved mild to moderate depression symptoms of women with menstrual disorder (P<0.02) after 6 months but not severe depressive symptoms. Overall significant improvement in anxiety (P<0.003) and depression (P<0.02) in IG in comparison to CG. |
| **Psycho-Biological Changes with Add on Yoga Nidra in Patients with Menstrual Disorders: a Randomized Clinical Trial. (Rani et al., 2016) [45]** | Randomised controlled trial    Active control group - No | Women with menstrual irregularities (n=87)  IG (n=45)  CG (n=42)  group. | Yoga Nidra therapy and pharmacotherapy  vs.  Pharmacotherapy | Anxiety Depression Positive well-being Self-control General health Vitality | PGWBI (Translated Hindi version) | India | Baseline, 6 months | IG improved depressive symptoms of women with menstrual disorder (P<0.02) after 6 months compared to CG. Significant improvement in domains of anxiety (P<0.01), depression (P<0.02), positive well-being (P<0.01), general health (P<0.04) and vitality (P<0.02) in IG was noted after six months of yogic intervention when compared to CG. |
| **The impact of an add-on video assisted structured aerobic exercise module on mood and somatic symptoms among women with depressive disorders: study from a tertiary care centre in India (Roy, ‎2018) [46]** | Randomised controlled trial.  Active control - no | Women inpatients (n=40) diagnosed with depressive disorder  IG: (n=20)  CG: (n=20) | Intervention group received a Video Assisted Structured Aerobic Exercise Program  vs. TAU | Reduction in depression | HDRS VAMS  DSSS | India | Baseline and post intervention. | IG versus CG improved levels of depression (P<0.05) at the programme’s end but not mood score or somatic symptoms related to depression. |
| **Cognitive-behavioural therapy for depression among menopausal woman: A randomized controlled trial. (Reddy et al., 2019) [47]** | Randomised controlled trial  Active control - no | Menopausal women (n= 102)  IG (n=51)  CG (n=51) | Six weekly group CBT sessions  vrs  TAU | Depression | CES-D | India | Baseline, post and 6-month. | Compared with the CG, the IG group showed statistically significant decrease in depression scores (P = 0.000) over the 6-month period. |
| **A randomized controlled effectiveness trial of cognitive behaviour therapy for post-traumatic stress disorder in terrorist-affected people in Thailand (Bryant et al., 2011) [59]** | Randomised controlled trial    Active control group - yes | (N=28) Survivors of terrorist attacks in southern Thailand  IG (n=16)  CG (n=12) | 8 sessions of either CBT vrs TAU (supportive counselling) | Reduction in PTSD symptoms | PSS-I  BDI  ICG | Thailand | Baseline, 8 weeks and 3 months | Compared with CG the IG reported significantly improved PTSD (P=0.001), depressive symptoms (P=0.004) and complex grief post treatment symptoms (P=0.001) post intervention and 3 months (P=0.007 for PTSD; P=0.003 for depression and P=0.003 for complicated grief) post treatment. |
| **Evaluation of a bibliotherapy manual for reducing psychological distress in people with depression: a randomized controlled trial. (Songprakun & McCann, 2012)a [61]** | Randomised controlled trial    Active control group - No | (N= 56) participants with moderate depression.  CG (n = 29) IG (n = 27) | 8-week self-help manual (bibliotherapy) plus standard care and treatment  vs. wait-list control (standard care and treatment) | Depression Psychological Distress | RS  CES-D  K-10 | Thailand | Baseline,  8 weeks, 12 weeks | IG versus CG improved depression post treatment (P=0.018) and 4 weeks (depression P=0.005) but no change in psychological distress. |
| **Effectiveness of a self-help manual on the promotion of resilience in individuals with depression in Thailand: a randomised controlled trial. (Songprakun & McCann, 2012)b [62]** | Randomised controlled trial    Active control group - No | (N= 56) participants with moderate depression. CG (n = 29) IG (n = 27) | 8-week self-help manual (bibliotherapy) plus standard care and treatment  vs. wait-list control (standard care and treatment). | Resilience Depression | RS  CES-D  K-10 | Thailand | 8 weeks, 12 weeks | IG versus CG improved resilience levels (P=0.029), and depression post treatment (P=0.018) and 4 weeks hence (resilience P=0.004; depression P=0.005). |
| **Evaluation of a cognitive behavioural self-help manual for reducing depression: a randomized controlled trial (Songprakun & McCann, 2012)c [63]** | Randomised controlled trial    Active control group - No | (N= 56) participants with moderate depression. CG (n = 29) IG (n = 27) | 8-week self-help manual (bibliotherapy) plus standard care and treatment  vs. wait-list control (standard care and treatment) | Depression | RS  CES-D  K-10 | Thailand | Baseline,  8 weeks, 12 weeks | IG group showed a signiﬁcant decrease in depression scores from baseline to post-test (P = 0.018) and from baseline to follow-up (P = 0.005), but no signiﬁcant decrease from post-test to follow-up (P= 1.00). |
| **Effectiveness of community-based depression intervention programme (ComDIP) to manage women with depression in primary care- randomised control trial. (Indu et al., 2018) [53]** | Randomised Controlled Trial  Active control group - no | Out-patient client (N=60) with moderate or severe depression Age: 18-60 years. | Community-based depression intervention programme (ComDIP) vs. TAU | Depression Quality of Life | HAM-D MADRS  SF-8  MINI Version 5  PHQ-9 | India | Baseline and at 8 weeks. | In the IG there was a large effect on depressive severity with women with depression (P = 0.01) and quality of life (P=0.006) was found at 8 weeks versus CG. |
| **Effectiveness of non-medical health worker-led counselling on psychological distress: a randomized controlled trial in rural Nepal. (Markkula et al., 2019) [65]** | Randomised Controlled Trial  Active control group - Yes | N=287, Age ≥16 years old Participants were randomized into two groups. Non-medical psychological and social counselling (PSY) group = (IG) 141, and Enhanced usual care (EUC) (CG) group = 146. | .  PSY was provided by lay counsellors.  vs. EUC was provided by trained primary health workers. | Depression  Anxiety | GHQ-12  BDI | Nepal | Baseline, 1 month and 6 months. | IG who received 6 months training, delivered in a primary care setting was effective versus CG at improving depressive and anxiety symptoms at 1 and 6 months (P-Values not reported). |
| **Brief cognitive behavioral therapy for depression among patients with alcohol dependence in Thailand (Thapinta et al., 2014) [48]** | Randomised controlled trial    Active control group - No | Eighty (N = 80) patients with alcohol dependence and depression  IG (n=40)  CG (n=40) | Brief six-session cognitive behavioural therapy  vs.  TAU | Depression Alcohol dependence | 8-question Suicidal Risk Scale  9-item self-administered depression scale (9Q). | Northern Thailand | Baseline, week 3, week 7 | IG delivered over a 3 week period was effective at reducing depression among Thai general hospital patients with alcohol dependence post intervention (P<.01) and 7 weeks later (P<.01). |
| **Effectiveness of a community-based intervention for people with schizophrenia and their caregivers in India (COPSI): a randomised controlled trial (Chatterjee et al., 2019) [55]** | Randomised Controlled Trial  Active control group - Yes | Out-patient client with Schizophrenia and their caregiver in three communities. N=282,  Age: 16-60 years old,  IG group (n= 187)  CG (n= 95) | Community care for People with Schizophrenia in India (COPSI) Vs Facility-based care (TAU). | Change in Schizophrenia symptoms and disabilities Adherence to antipsychotic treatment and experience of stigma and discrimination. | PANSS  IDEAS | India | Baseline, 6 months and 12 months. | For the IG in the intensive engagement (0-3months), the stabilization (4-7 months), and the maintenance (8-12 months) vs CG reduced positive and negative symptoms of Schizophrenia and disabilities at 12 months (P=0.01). |
| **The effect of telephone support on depressive symptoms among HIV-infected pregnant women in Thailand: an embedded mixed methods study (Ross et al., 2013) [60]** | Randomised controlled trial.  Active control - no | (N=40) pregnant HIV infected women with depressive symptoms | Weekly telephone support vrs TAU | Reduction in depressive symptoms | CES-D | Thailand | Baseline, 1 month and 2 months | Compared to CG the IG reduced depressive symptoms after 1 (P=0.044) and 2 months (P=0.001) of receiving this intervention. |
| **Efficacy and cost-effectiveness of drug and psychological treatments for common mental disorders in general health care in Goa, India: a randomised, controlled trial (Patel et al., 2003) [54]** | Randomised Controlled Trial  Active control group - Yes | Adult Out-patient client who came to get treatment at the district general hospitals (N=450)  Antidepressant Group (n=150), Placebo Group (n=150) and Psychological therapy Group (n=150) | Antidepressant or Placebo (IG) Vs Psychological Intervention (CG) | Psychiatric morbidity | GHQ  CIS-R. | India | Baseline, 2 months, 6 months and 12 months. | IG had reduced psychiatric morbidity (measure by CISR total score) than with placebo at 2 months (P=0.005) but not from 2 to 12 months (P=0.1). CG was not more effective than placebo for any outcome at all the time points (P=0.86 at 2 months and P=0.48 at 2-12 months). |
| **Resource activation for treating post-traumatic stress disorder, co-morbid symptoms and impaired functioning: a randomized controlled trial in Cambodia (Steinert et al., 2017) [66]** | Randomised Controlled Trial  Active control group - No | Out-patient clients (N=86) –  (IT n=53)  CT (n=33)  Age: ≥18 years | ROTATE (Resource-oriented trauma therapy combined with eye movement desensitization and reprocessing (EMDR) resource installation.  vs.  TAU | Remission rates of PTSD Symptoms of depression and anxiety Self-perception of functioning (SPF)  Depression and emotional distress status. | PCL-C  HTQ  HSCL-25 | Cambodia | Baseline, post intervention | IG reported significantly reduced levels of depression, anxiety and impaired functioning and increased PTSD remission rates (P<0.001) compared with CG. |
| **The Effectiveness of Eye Movement Desensitization and Reprocessing Therapy to Treat Symptoms Following Trauma in Timor Leste. (Schubert et al., 2016) [69]** | Randomised controlled trial    Active control group - no | Out-patient clients (N=21), Age: 18-65 years  Symptom changes post-EMDR treatment were  compared to a stabilization control intervention period in which participants served as their own waitlist control. | EMDR therapy  vs. TAU | Severity of PTSD, Depression and Anxiety symptoms: Psychophysiological response | HTQ,  HSCL-25  SUDs  VAS | Timor Leste | Baseline, post intervention, 3 months | IG versus CG control was significant in reducing depression, anxiety and PTSD post intervention (P<0.001), and depression three months later (P=0.034). There were no significant differences in anxiety or PTSD symptoms 3 months later. |
| **Unanticipated effect of a randomized peer network intervention on depressive symptoms among young methamphetamine users in Thailand (German et al., 2012) [57]** | Randomised Controlled Trial  Active control group - Yes | Methamphetamine (MA) Users (Index Participant) and their sex partners/drug using friends (network members) in community. N=983  IG=495  CG=488 Age: 18-25 years, | Peer-educator network-oriented intervention vs.  Standard best practice, known as life skill intervention in group setting. | Sexual risk behaviour  MA use Depression | CES-D was used to measure depressive symptoms. | Thailand | Baseline, 3 months, 6 months and 12 months. | Post-assessment the IG group showed a significantly different decreasing trend in depressive symptoms as indicated by the condition-by-time interaction (P < 0.0001).  Symptoms scores of PTSD and anxiety were remained the same at 3-month follow-up, but depression scores continued to decrease. |
| **Effect of mobile phone-based psychotherapy in suicide prevention: a randomized controlled trial in Sri Lanka (Marasinghe et al., 2012) [70]** | Randomised controlled trial  Active control - no | Participants admitted to hospital after self-harm and showing suicidal intent (n=68)  Age: 15–74 years  IG (n=34)  CG (n=34) | BMT Group (Meditation Problem solving interventions to increase social support, alcohol and other drug use and training to use mobile phones.  Vs. TAU (D-BMT) group received the intervention at six months post-hospitalization. | Unclear. Paper states - 'improve outcomes relative to usual care among suicide attempters' | BSS  BDI, BDI-1A, BDI-I  MOS social support survey AUDIT | Sri Lanka | Baseline, 3 months, 6 months | IG reported reduced suicidal ideation and depression in people who recently attempted suicide versus CG at 6 and 12 months (No P-Values reported). |
| **Testimony Therapy With Ritual: a Pilot Randomized Controlled Trial (Esala & Taing, 2017) [67]** | Randomised Controlled Trial  Active control group - No | Samples were selected from Khmer Rouge torture survivors (N=120)  IG (n= 60)  CG (n= 60) | Testimony Therapy plus ceremony (culturally adapted ceremony which involves a Buddhist ceremony and a truth-telling event) vs.  TAU | Severity of PTSD, anxiety, and depression | PCL-C HSCL-25 | Cambodia | Baseline, 3 months and 6 months. | Compared to the CG the IG reported significant reduction in depression symptoms (P= 0.001, but symptoms did not significantly decrease from 3 months to 6 months. Again, there was a significant reduction in anxiety symptoms for IG from baseline to 3 months (P = 0.001), but symptoms did not significantly decrease from 3 months to 6 months. |
