# Supplementary material for: Psychological and social interventions for mental health issues and disorders in Southeast Asia: a systematic review
Source: Int J Ment Health Syst. 2021 Jun 5;15:56. doi: 10.1186/s13033-021-00482-y (PMC8178881; doi:10.1186/s13033-021-00482-y)
Supplement: Supplementary file 2 — Additional file 2. Cochrane Risk of Bias Tool for RCTs. [file 13033_2021_482_MOESM2_ESM.docx]

Additional File 2: Cochrane Risk of Bias Tool for RCTs

| **Title of review & Authors** | **Type of bias** | **Selection** | **Performance** | **Detection** | **Attrition** | **Reporting** | |
| --- | --- | --- | --- | --- | --- | --- | --- |
|  | **Random sequence generation** | **Allocation concealment** | **Blinding of participants and personnel** | **Blinding of outcome assessment** | **Incomplete outcome data** | **Selective reporting** | **Other biases** |
| **Lay delivered interventions or programmes** | | | | | | | |
| **Patel et al., 2017 [49]** | Low | Low | Moderate | Low | Low | Low | Low |
| **Vanobberghen et al., 2020 [71]** | Low | Low | Moderate | Low | Moderate | Moderate | Low |
| **Patel et al., 2010 [56]** | Low | Low | Moderate | Low | Low | Low | Moderate |
| **Patel et al., 2011 [40]** | Low | Low | Moderate | Moderate | Low | Low | Moderate |
| **Fuhr et al., 2019 [41]** | Low | Low | Moderate | Low | Low | Low | Moderate |
| **Arjadi et al., 2018 [68]** | Low | Low | Low | Low | High | Low | Low |
| **Jordans et al., 2019 [64]** | Low | High | High | High | Low | Low | Moderate |
| **Yoga, aerobic and/or meditation based interventions or programmes** | | | | | | | |
| **Tolahunase et al., 2018a [42]** | Low | Moderate | High | High | Low | Low | Moderate |
| **Siritienthong et al., 2018 [58]** | High | Unclear | High | Unclear | High | Low | High |
| **Tolahunase et al., 2018b [51]** | Low | Low | High | High | Low | Low | Moderate |
| **Rentala et al., 2013 [50]** | Moderate | High | High | High | Low | Low | Moderate |
| **Rentala et al., 2015 [43]** | Low | High | High | High | Low | Low | Moderate |
| **Rani et al., 2011[44]** | Low | High | High | High | High | Low | High |
| **Rani et al., 2012 [52]** | Low | Low | High | High | Low | Low | Moderate |
| **Rani et al., 2016 [45]** | Low | High | High | High | High | Low | High |
| **Roy, ‎2018 [46]** | High | High | High | High | High | High | High |
| **CBT oriented interventions or programmes** | | | | | | | |
| **Reddy et al., 2019 [47]** | Unclear | High | High | Unclear | High | High | High |
| **Bryant et al., 2011 [59]** | Low | Low | High | High | Moderate | Unclear | High |
| **Songprakun & McCann, 2012a [61]** | Low | High | High | High | Low | Low | Moderate |
| **Songprakun & McCann, 2012b [62]** | Low | High | High | High | Low | Low | Moderate |
| **Songprakun & McCann, 2012c [63]** | Low | High | High | High | Low | Low | Moderate |
| **Indu et al., 2018 [53]** | Low | Low | Low | Low | Low | High | Low |
| **Markkula et al., 2019 [65]** | Moderate | Moderate | Moderate | Moderate | High | High | Moderate |
| **Thapinta et al., 2014 [48]** | High | High | High | High | Low | Low | Moderate |
| **Health worker delivered interventions or programmes** | | | | | | | |
| **Chatterjee et al., 2019 [55]** | Low | Low | Low | Low | High | High | Moderate |
| **Ross et al., 2013 [60]** | High | High | High | High | Low | Unclear | High |
| **Patel et al., 2003 [54]** | Low | Low | Low | Low | High | High | Low |
| **EMDR based interventions or programmes** | | | | | | | |
| **Steinert et al., 2017 [66]** | Low | Moderate | Moderate | Low | High | Moderate | Low |
| **Schubert et al., 2016 [69]** | High | High | Moderate | Moderate | High | High | Moderate |
| **Hybrid Programmes** | | | | | | | |
| **German et al., 2012 [57]** | Low | Low | High | High | High | High | Moderate |
| **Marasinghe et al., 2012 [70]** | High | High | High | Moderate | Low | Unclear | High |
| **Esala & Taing, 2017 [67]** | High | High | Moderate | Low | Low | Moderate | High |
